# *De novo* synthesis of short‐chain aldehydes and hydrocarbons secreted by the brown marmorated stink bug *Halyomorpha halys*

**DOI:** 10.1002/2211-5463.70314

**Published:** 2026-07-21

**Authors:** Haruna Fujimori, Koji Noge

**Affiliations:** ^1^ Department of Biological Production Akita Prefectural University Japan; ^2^ Present address: Division of Cancer Stem Cell Miyagi Cancer Center Research Institute Natori Miyagi Japan

**Keywords:** (*E*)‐2‐decenal, 4‐oxo‐(*E*)‐2‐hexenal, biosynthesis, dodecane, fatty acids, heteroptera, tridecane

## Abstract

Most heteropterans release pungent or characteristic secretions that help protect them against predators and pathogens. However, the biosynthesis of these secretions is poorly understood. The secretion of the brown marmorated stink bug *Halyomorpha halys* (Hemiptera: Pentatomidae) mainly comprises 4‐oxo‐(*E*)‐2‐hexenal, (*E*)‐2‐decenal and tridecane, a mixture representative of that released by many heteropterans. Here, we used a feeding assay demonstrated to show that the secretory components of *H*. *halys* contained ^13^C atoms derived from ^13^C‐labelled glucose. Thus, we show that *H*. *halys* can synthesise myristic, palmitic, stearic, palmitoleic and oleic acids *de novo*, but not linoleic acid. These results suggest that the secretory components of *H*. *halys* are synthesised from acetyl‐CoA generated through glycolysis or chain shortening of saturated fatty acids via β‐oxidation pathway.

## Graphical abstract

The brown marmorated stink bug, *Halyomorpha halys*, mainly secretes 4‐oxo‐(*E*)‐2‐hexenal, (*E*)‐2‐decenal, dodecane and tridecane, a mixture representative of that released by many heteropterans. We demonstrated that *H*. *halys* can synthesise these secretory components *de novo* using dietary glucose.

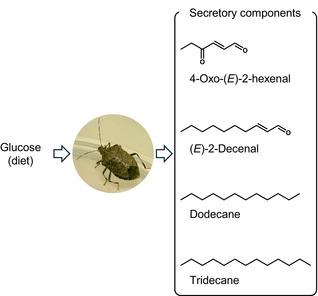

**Abbreviations**
DAG: dorsal abdomen gland
GC: gas chromatography
MS: mass spectrometry
MTG: metathoracic scent gland
OHE: 4‐oxo‐(*E*)‐2‐hexenal

Insects adaptively utilise chemicals acquired by *de novo* synthesis or uptake from their diet for communication and defence [1, 2, 3, 4]. Heteropteran insects produce a variety of chemicals in their secretory glands, which are located on the dorsal side of the abdomen (DAGs; dorsal abdomen glands) in nymphs and on the ventral side of the thorax (MTGs; metathoracic scent glands) in adults [5, 6, 7]. The secretions of heteropteran insects function as pheromonal signals between conspecific individuals and as defensive substances against predatory insects, vertebrates and microbes [5, 6, 7, 8, 9, 10, 11, 12, 13]. These secretions have been well studied and mainly comprise short‐chain saturated/unsaturated aldehydes, alcohols, esters and sometimes alkanes; however, their synthesis has attracted much less attention. For example, *n*‐hexyl acetate is converted into hexanal via 1‐hexanol by esterases and alcohol dehydrogenases found in the scent glands of two coreid bug species [14, 15]. However, the synthesis of the *n*‐hexyl acetate carbon chain remains unclear. Gordon et al. [16] reported that ^14^C‐labelled sodium acetate was incorporated into three secretory components [(*E*)‐2‐hexenal, (*E*)‐2‐decenal and tridecane] of the green vegetable bug *Nezara viridula*. This implies that short‐chain aldehydes in heteropteran secretions may be synthesised *de novo* via the condensation of two‐carbon acetyl units or generated as degradation products of fatty acids. It is also reported that the reduction in the production of (*E*)‐2‐hexenal and 4‐oxo‐(*E*)‐2‐hexenal (OHE) in *Thasus neocalifornicus* nymphs after antibiotic treatment correlates with a reduction in the levels of the symbiotic *Wolbachia* bacterium in their DAGs [17]. However, it remains unclear whether this symbiont is involved in the production of these secretory components.

*Halyomorpha halys* (Stål) (Hemiptera: Heteroptera: Pentatomidae) is a polyphagous plant pest distributed initially in subtropical and temperate areas of East Asia (Japan, China and Korea) and is now found in North America, South America and Europe [18, 19, 20]. The insect secretions mainly consist of OHE, (*E*)‐2‐decenal and tridecane (Fig. 1A) [21, 22, 23]. Dodecane, an even‐chain hydrocarbon, was detected as a minor component of *H*. *halys* secretions (Fig. 1A). In most cases, heteropteran nymphs and adults have different secretory glands, resulting in different semiochemical phenotypes between developmental stages [15]. In the case of *H*. *halys*, these four components do not differ between nymphs and adults, suggesting that they are synthesised by the same or similar biosynthetic systems in both nymphs and adults. For these reasons, and because *H*. *halys* can be reared easily on fresh peanut seeds or carrots in the laboratory, this insect is considered a suitable model for studying whether short‐chain aldehydes and hydrocarbons are synthesised *de novo*.

**Fig. 1:**
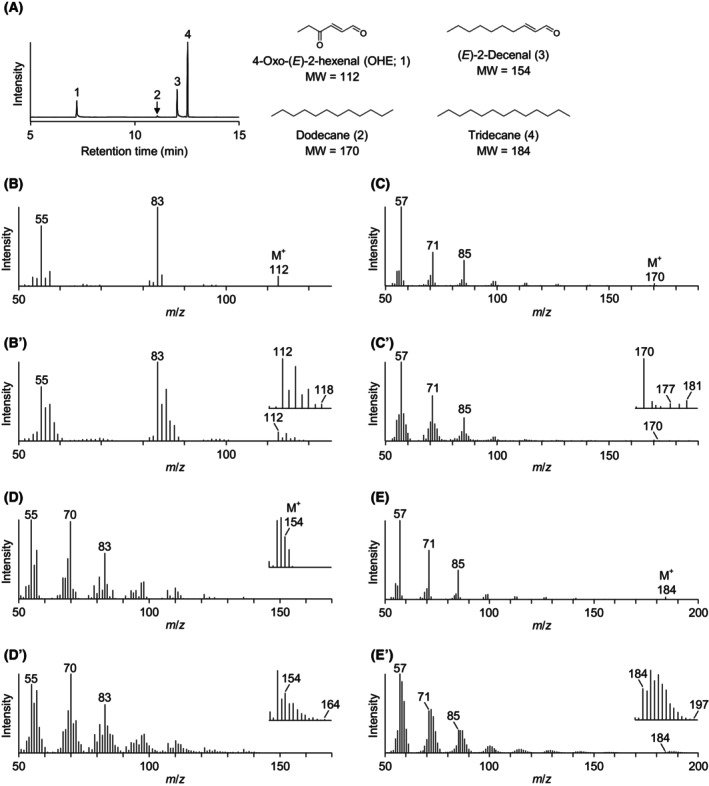
Gas chromatography/mass spectrometry (GC/MS) analysis of secretions in natural and ^13^C‐labelled glucose‐fed *Halyomorpha halys*. (A) Typical gas chromatogram of the secretion of natural *H*. *halys* and chemical structure of its components. Peak 1: 4‐oxo‐(*E*)‐2‐hexenal (OHE); Peak 2: dodecane; Peak 3: (*E*)‐2‐decenal; and Peak 4: tridecane (B–E) Mass spectra of the natural components of *H*. *halys*. (B) OHE. (C) Dodecane. (D) (*E*)‐2‐Decenal. (E) Tridecane. (B′–E′) Mass spectra of ^13^C‐labelled glucose‐fed *H*. *halys* secretion. (B′) OHE reared using ^13^C‐labelled glucose. (C′) Dodecane reared with ^13^C‐labelled glucose. (D′) (*E*)‐2‐decenal reared with ^13^C‐labelled glucose. (E′) Tridecane reared with ^13^C‐labelled glucose.

In this study, ^13^C‐labelled glucose, likely present in diet of herbivorous stink bug, was used to investigate the synthesis of secretory components and fatty acids in *H*. *halys*. The fatty acid composition of *H*. *halys* gland extract was analysed and compared with that of the diet (fresh peanut seeds and carrots) to determine the metabolic origin of the secretory components of *H*. *halys*.

## Materials and methods

### Chemicals

Fully ^13^C‐labelled d‐Glucose (99%) was purchased from Cambridge Isotope Laboratories, Inc. (Andover, MA, USA). (*E*)‐2‐Decenal, methyl myristate, methyl stearate, methyl palmitoleate (methyl *cis*‐9‐hexadecenoate) and methyl linoleate were purchased from Tokyo Chemical Industry Co., Ltd. (Tokyo, Japan). Dodecane, 1‐dodecene, tridecane, methyl palmitate and methyl oleate were purchased from FUJIFILM Wako Pure Chemical Corporation (Osaka, Japan). Methyl α‐linolenate and methyl γ‐linolenate were purchased from Sigma‐Aldrich (St. Louis, MO, USA). Potassium sorbate and agar powder were purchased from the Kanto Chemical Co. (Tokyo, Japan). OHE was synthesised according to the method described by Moreira and Millar [24]. High‐analytical‐grade and HPLC‐grade solvents were used in this study.

### Insects and feeding experiments

*Halyomorpha halys* nymphs were purchased from Sumika Technoservice Corporation (Takarazuka, Japan). The nymphs were fed fresh peanut seeds and water and maintained at 25 ± 2 °C. After eclosion, the adults were reared in the same manner as the nymphs until fatty acid analysis.

For the feeding assays, a carrot‐based artificial diet containing fully ^13^C‐labelled d‐glucose (final concentration approximately 20 mg/mL) was prepared. A carrot paste was prepared by mixing grated carrot and water in a ratio of approximately 5:1. 400 mg of fully ^13^C‐labelled d‐glucose and 20 mg of potassium sorbate were placed in a 30‐mL beaker, and the carrot paste was added up to the 10 mL mark. They were mixed thoroughly to be homogeneous. Agar powder was dissolved in hot water [1.4% (w/v)], and the 10 mL of the resulting agar solution was mixed with the above carrot‐based mixture. The mixture was set at 4 °C and kept as is before use.

The fourth‐instar nymphs kept at 25 ± 2 °C and fed the carrot‐based artificial diet containing ^13^C‐labelled glucose for 3–4 weeks until they reached the adult stage. In a preliminary experiment, a few nymphs fed only the carrot‐based artificial diet did not fully eclose; therefore, fresh peanut seeds were added to their diet once a week. Following the moult to the fifth instar, they emerged as adults within 2–3 weeks. After adult emergence, the adults were used for the fatty acid analysis. Their fresh exuviae from the fifth‐instar nymphs were used for the chemical analysis of secretions because the nymphal secretions remained on the exuviae when the nymphs ecdysed to adults [15].

### Chemical analysis of secretion from ^13^C‐labelled glucose‐fed *H*. *halys*

Each fresh exuvium was extracted using 0.5 mL of hexane for 5 min as a source of nymphal secretion. An aliquot of the hexane extract (1.0 μL) was analysed via gas chromatography/mass spectrometry (GC/MS) equipped with a DB‐5MS capillary column (30 m × 0.25 mm i.d., 0.25 μm film thickness; Agilent Technologies, Inc., Santa Clara, CA, USA). GC/MS analysis was performed using a Clarus 600 GC/MS (PerkinElmer Inc., Shelton, CT, USA) operated at 70 eV with helium as the carrier gas at 1.0 mL/min flow rate. The oven temperature was maintained at 50 °C for 3 min, then increased to 290 °C at a rate of 10 °C/min and held at 290 °C for 5 min. The injector and detector temperatures were maintained at 250 °C. The secretory components [OHE, (*E*)‐2‐decenal, dodecane and tridecane] were identified by comparing their GC retention times with those of commercial and synthesised standards. Extracts of the exuvium obtained from more than six individuals were analysed.

The incorporation of ^13^C atoms into the secretory components of *H*. *halys* fed on ^13^C‐labelled glucose was evaluated by comparing the proportion of secretory components containing isotopes with the proportion of those containing naturally occurring isotopes (^13^C or ^18^O). The following diagnostic ions were used to calculate the proportion of secretory components containing naturally occurring isotopes: mass‐to‐charge ratio (*m/z*) 112 (M^+^), 113 and 114 for OHE; *m/z* 136 ([M − H_2_O]^+^), 137 and 138 for (*E*)‐2‐decenal; *m/z* 57 (C_4_H_9_^+^) and 58 for dodecane; and *m/z* 184 (M^+^), 185 and 186 for tridecane. The sum of the peak areas of the isotopic ions was divided by the sum of the peak areas of all diagnostic ions to calculate the proportion of the secretory components containing naturally occurring isotopes. The following ions were used to calculate the proportion of secretory components derived from ^13^C‐labelled glucose‐fed *H*. *halys*: *m/z* 112–118 for OHE, *m/z* 136–146 for (*E*)‐2‐decenal, *m/z* 57–61 for dodecane and *m/z* 184–197 for tridecane. Similarly, the proportion of the secretory components containing isotopes in ^13^C‐labelled glucose‐fed *H*. *halys* was calculated. All data were analysed with the Wilcoxon rank sum test in JMP 5.1.2 [25].

### Fatty acid analysis in the MTG complex of *H*. *halys*

Adult ^13^C‐labelled glucose‐fed *H*. *halys* were kept at −20 °C for 30 min, and the metathoracic scent gland (MTG) complex was then removed via dissection (Fig. S1). The MTG complex was removed and homogenised in 2.1 mL chloroform/methanol (2 : 1, v/v). The mixture was left to stand overnight at room temperature (25 ± 2 °C), and the debris was removed via filtration through cotton. A portion of the extracts (1.0 mL) was washed two times with water and concentrated to dryness under nitrogen gas. The residues were dissolved in 1.0 mL of 0.5 m HCl/methanol and heated at 100 °C overnight to obtain fatty acid methyl esters after the hydrolysis. The reaction mixtures were concentrated to dryness under nitrogen gas, dissolved in 1.0 mL of chloroform containing 20 ng/μL 1‐dodecene as an internal standard and aliquots (1.0 μL) were analysed using GC/MS (*n* = 5). GC/MS analysis was performed using the same instrument described above; however, it was equipped with a DB‐23 capillary column (30 m × 0.25 mm i.d., 0.15‐μm film thickness; Agilent Technologies, Inc.). The following oven temperature program was used: 50 °C for 1 min, then increased to 175 °C at 25 °C/min, then to 215 °C at 4 °C/min and held at 215 °C for 5 min. The injector and detector temperatures were maintained at 250 °C. Fatty acid methyl esters were identified by comparing their GC retention times with those of commercial standards.

Adult *H*. *halys* fed peanut seeds, but not ^13^C‐labelled glucose, were dissected as described above, and the MTG complex was removed. Its fatty acid composition was analysed using the procedure described above (*n* = 6 each).

The fatty acids present in fresh peanut seeds and carrots (diet of *H*. *halys*) were analysed. Fatty acids were extracted from half of the fresh peanut seeds (0.3–0.4 g) or a piece of carrot (ca. 1 g) and analysed using the same procedure described above (*n* = 3 each).

The peak area of each fatty acid methyl ester was calculated using the following ions: *m/z* 55 for methyl oleate and the internal standard (1‐dodecene); *m/z* 67 for methyl linoleate; *m/z* 74 for methyl myristate, methyl palmitate, and methyl stearate; and *m/z* 79 for methyl α‐linolenate. The concentration of fatty acids was quantified by comparing the relative peak area ratio of the fatty acid methyl ester to the internal standard with that of the calibration standard (2.5–20 ng/μL for methyl myristate and 12.5–125 ng/μL for the others). The composition ratios of these six fatty acids were calculated, and the ratios in the *H*. *halys* MTG complex and diets were analysed with one‐way ANOVA followed by the Tukey–Kramer test with JMP 5.1.2 [25].

## Results

### Incorporation of ^13^C‐labelled glucose into *H*. *halys* secretions

GC/MS analysis demonstrated that two aldehydes and two hydrocarbons extracted from the fresh exuviae (= nymphal secretions) contained ^13^C atoms derived from ^13^C‐labelled glucose (*n* = 6; Fig. 1B–E,B′–E′). The OHE present in the secretions of *H*. *halys* fed ^13^C‐labelled glucose showed ions at *m/z* 118 (M^+^ + 6), along with its natural molecular ion at *m/z* 112 (Fig. 1B,B′). This indicates that ^13^C atoms were incorporated into the OHE. The proportion of the OHE containing isotopes in ^13^C‐labelled glucose‐fed *H*. *halys* was calculated to be 47.7 ± 8.6% (mean ± SE; *n* = 6). The value was significantly higher than that observed in natural OHE (3.1 ± 1.8%; *P* < 0.05). In addition, (*E*)‐2‐decenal present in ^13^C‐labelled glucose‐fed *H*. *halys* showed ions at *m/z* 164 (M^+^ + 10), together with its natural molecular ion at *m/z* 154 (Fig. 1D,D′), thereby indicating the incorporation of ^13^C atoms. The proportion of the (*E*)‐2‐decenal containing isotopes in ^13^C‐labelled glucose‐fed *H*. *halys* was calculated to be 44.4 ± 8.5% (mean ± SE; *n* = 6). The value was significantly higher than that observed in natural (*E*)‐2‐decenal (3.6 ± 2.8%; *P* < 0.05). Similarly, the tridecane present in ^13^C‐labelled glucose‐fed *H*. *halys* gave rise to ions at *m/z* 197 (M^+^ + 13), indicating that ^13^C atoms were incorporated into tridecane (molecular ion: *m/z* 184; Fig. 1E,E′). The proportion of the tridecane containing isotopes in ^13^C‐labelled glucose‐fed *H*. *halys* was calculated to be 62.2 ± 12.2% (mean ± SE; *n* = 6). The value was significantly higher than that observed in natural tridecane (6.9 ± 2.6%; *P* < 0.05). Considering that the amount of dodecane in the *H*. *halys* secretion was small compared to that of the other three components (Fig. 1A), determining the incorporation of ^13^C atoms based on the molecular ion was challenging. The incorporation of ^13^C‐labelled glucose into dodecane was confirmed by analysing the dodecane fragment ions in ^13^C‐labelled glucose‐fed *H*. *halys*. In the mass spectrum of natural dodecane, a fragment ion (base ion) at *m/z* 57 corresponding to C_4_H_9_^+^ was observed (Fig. 1C). Dodecane present in ^13^C‐labelled glucose‐fed *H*. *halys* showed ions at *m/z* 61, together with the naturally occurring *m/z* 57 (Fig. 1C′). This indicates that these fragment ions contained ^13^C atoms. From these fragment ions, the proportion of the dodecane containing isotopes in ^13^C‐labelled glucose‐fed *H*. *halys* was estimated to be 35.2 ± 9.1% (mean ± SE; *n* = 6). The value was significantly higher than that observed in natural dodecane (1.5 ± 1.3%; *P* < 0.05).

### Fatty acid content in *H*. *halys* MTG complex, midgut and diet

The fatty acid methyl esters isolated from the MTG complex of adult *H*. *halys* were methyl myristate (0.4 ± 0.3 μg/individual), methyl palmitate (33.1 ± 10.7 μg/individual), methyl stearate (13.1 ± 2.0 μg/individual), methyl oleate (74.7 ± 23.8 μg/individual) and methyl linoleate (46.6 ± 17.2 μg/individual). α‐Linolenic acid was not detected in the MTG complex under these conditions; however, it was detected in peanut seeds and carrots (Table 1). Peanut seeds contained methyl α‐linolenate together with five fatty acid methyl esters found in *H*. *halys*. Carrots contained methyl palmitate, methyl stearate, methyl oleate, methyl linoleate and methyl α‐linolenate; however, methyl myristate was not detected under these conditions. The composition of fatty acid methyl esters derived from food sources differed from that derived from the MTG complex of *H*. *halys*, suggesting that the fatty acid composition of *H*. *halys* secretions does not simply reflect the composition of the food consumed (Table 1).

**Table 1:** Fatty acid composition of *Halyomorpha halys* and its diet. Each compound was analysed as a methyl ester derivative. Composition ratio followed by the same letter within a row is not significantly different among samples (*P* > 0.05, Tukey–Kramer test). MTG, metathoracic scent gland; n.d., not detected.

Fatty acid	Composition (mean ± SE, %)		
	MTG complex	Peanut seed	Carrot
Myristic acid	0.1 ± 0.1^a^	0.01 ± 0.0^a^	n.d.
Palmitic acid	20.1 ± 0.9^a^	5.5 ± 0.2^b^	21.9 ± 0.7^a^
Stearic acid	9.8 ± 1.6^a^	2.4 ± 0.3^b^	4.0 ± 0.5^b^
Oleic acid	43.4 ± 2.2^a^	72.0 ± 1.8^b^	10.6 ± 0.2^c^
Linoleic acid	26.5 ± 1.3^a^	20.1 ± 1.8^b^	59.3 ± 0.9^c^
α‐Linolenic acid	n.d.	0.03 ± 0.0^a^	4.3 ± 0.2^b^

### Incorporation of ^13^C‐labelled glucose into fatty acids in *H*. *halys*

**Fig. 2:**
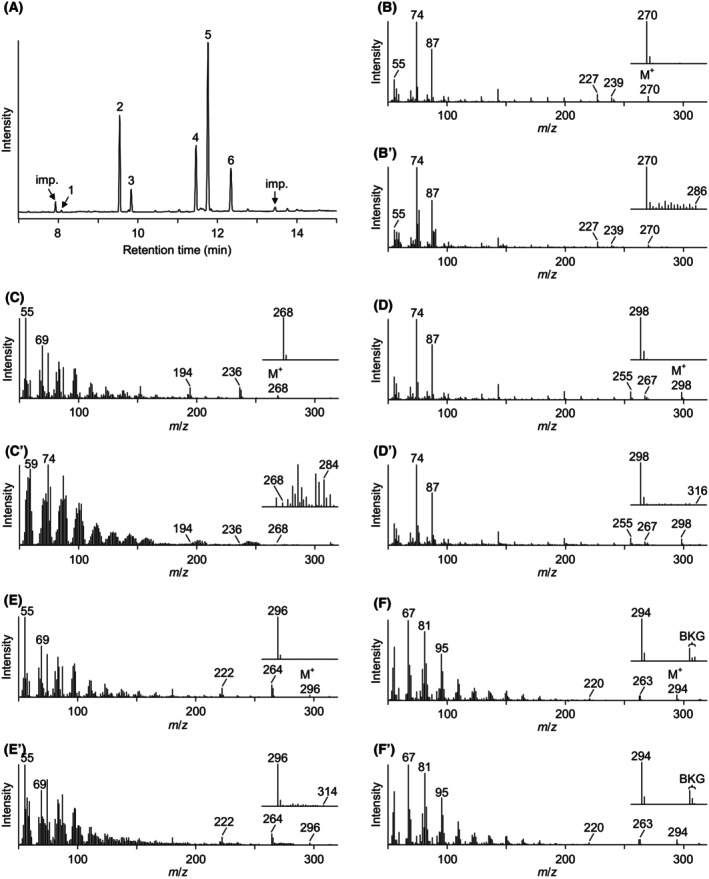
Gas chromatography/mass spectrometry (GC/MS) analysis of fatty acid derivatives in the metathoracic scent gland (MTG) complex of *Halyomorpha halys* reared with ^13^C‐labelled glucose. (A) Typical gas chromatogram of fatty acid derivatives in the MTG complex of *H*. *haly* reared with ^13^C‐labelled glucose. Peak 1: methyl myristate; Peak 2: methyl palmitate; Peak 3: methyl palmitoleate; Peak 4: methyl stearate; Peak 5: methyl oleate; Peak 6: methyl linoleate; imp.: impurity (B–F) Mass spectra of standard fatty acid methyl esters. (B) Methyl palmitate. (C) Methyl palmitoleate. (D) Methyl stearate. (E) Methyl oleate. (F) Methyl linoleate. BKG: background ions. (B′–F′) Mass spectra of fatty acid derivatives in the MTG complex of *H*. *haly* reared with ^13^C‐labelled glucose. (B′) Methyl palmitate reared with ^13^C‐labelled glucose. (C′) Methyl palmitoleate reared with ^13^C‐labelled glucose. (D′) Methyl stearate reared with ^13^C‐labelled glucose. (E′) Methyl oleate reared with ^13^C‐labelled glucose. (F′) Methyl linoleate reared with ^13^C‐labelled glucose.

The fatty acids biosynthesised in *H*. *halys* were identified using GC/MS of the corresponding fatty acid methyl ester derivatives (Fig. 2A). ^13^C atoms were incorporated into three saturated fatty acid methyl esters (methyl myristate, methyl palmitate and methyl stearate) (Figs 2B,D,B′,D′, and S2) and two monounsaturated fatty acid methyl esters (methyl palmitoleate and methyl oleate) (Fig. 2C,E,C′,E′), but not methyl linoleate (Fig. 2F,F′).

The mass spectrum of methyl palmitate is shown in Fig. 2 as a typical example of the incorporation of ^13^C‐labelled glucose into saturated fatty acid methyl esters. Natural methyl palmitate exhibits characteristic ions at *m/z* 74, 87 and 239, together with its molecular ion at *m/z* 270 (Fig. 2B). The molecular ion of methyl palmitate obtained from ^13^C‐labelled glucose‐fed *H*. *halys* exhibited ions at *m/z* 286 (M^+^ + 16), indicating the incorporation of ^13^C atoms (Fig. 2B′).

Similarly, the molecular ions arising from methyl stearate originating from ^13^C‐labelled glucose‐fed *H*. *halys* comprised those at *m/z* 316 (M^+^ + 18), indicating the incorporation of ^13^C atoms (Fig. 2D,D′).

The amount of the methyl myristate in *H*. *halys* MTG complex was lower than that of the other fatty acid methyl esters. The fragment ions observed at *m/z* 225 (corresponding to the loss of OCH_3_, a naturally occurring ion at *m/z* 211), indicating that ^13^C atoms were incorporated into myristic acid moieties (Fig. S2A,A′). The molecular ion of methyl myristate obtained from ^13^C‐labelled glucose‐fed *H*. *halys* exhibited an ion at *m/z* 256 (M^+^ + 14), together with the naturally occurring *m/z* 242 (Fig. S2B,B′).

The molecular ions of the two natural monounsaturated fatty acid methyl esters (methyl palmitoleate and methyl oleate) were *m/z* 268 and 296, respectively (Fig. 2C,E). When ^13^C‐labelled glucose was fed to *H*. *halys*, molecular ions corresponding to methyl palmitoleate and methyl oleate at *m/z* 284 (M^+^ + 16) and 314 (M^+^ + 18) were observed, respectively (Fig. 2C′,E′). These results indicate the incorporation of ^13^C atoms. In contrast, the molecular ion of linoleic acid methyl ester (*m/z* 294) and its mass spectrum did not change with or without the uptake of ^13^C‐labelled glucose (Fig. 2F,F′).

## Discussion

The results showed the incorporation of ^13^C‐labelled glucose into the four components of *H*. *halys* nymphal secretion: OHE, (*E*)‐2‐decenal, dodecane and tridecane. This suggests that, during glycolysis, ^13^C‐labelled glucose is first metabolised to ^13^C‐labelled acetyl‐CoA via pyruvate, and the resulting acetyl‐CoA is then incorporated into these secretory components.

In this study, myristic, palmitic, stearic, palmitoleic and oleic acids were synthesised *de novo* from ^13^C‐labelled glucose in *H*. *halys*, whereas linoleic acid was not. These results are consistent with those of other heteropteran species. Cripps et al. [26] reported that three heteropteran species (*Lygus hesperas*, *Lygaeus kalmii* and *Oncopeltus fasciatus*) do not synthesise linoleic acid *de novo*. Although some insects and mites can synthesise linoleic acid *de novo* [27, 28, 29, 30], *H*. *halys* lacks the Δ12‐desaturase that converts oleic acid into linoleic acid. Hence, similar to other animals that require polyunsaturated fatty acids as essential nutrients from food, *H*. *halys* cannot synthesise α‐linolenic acid from linoleic acid (Fig. 2).

OHE is present in volatiles from damaged plant leaves and peroxidised vegetable oil and is thought to be generated through oxidative cleavage of the carbon–carbon bond in α‐linolenic acid [31, 32]. However, because *H*. *halys* can synthesise OHE from ^13^C‐labelled glucose, *H*. *halys* may utilise a different route for OHE synthesis from a starting material other than α‐linolenic acid.

The incorporation of ^14^C‐labelled sodium acetate into (*E*)‐2‐hexenal has been observed in *N*. *viridula* [16] and the Florida wood cockroach *Eurycotis floridana* [33]. The incorporation of ^13^C‐labelled glucose into (*E*)‐2‐decenal in *H*. *halys* secretions was similar to that observed in previous studies [16, 33], as dietary glucose is metabolised to acetyl‐CoA. In contrast, 2‐decenal, with an unspecified geometry, has been reported as a degradation product of oxidised oleic acid [34]. Thus, the possibility that ^13^C‐labelled (*E*)‐2‐decenal is produced via the oxidation of ^13^C‐labelled oleic acid cannot be ruled out in *H*. *halys*. Future research will explore how exogenous isotope‐labelled fatty acids are metabolised in *H*. *halys*.

Saturated and/or unsaturated aldehydes, such as hexanal, (*E*)‐2‐hexenal, (*E*)‐2‐octenal and (*E*)‐2‐decenal, are often found in heteropteran secretions. Although hexanoic acid has been detected in adult *Hygia lativentris* in amounts proportional to the age of the insect [15], carboxylic acids, which are the oxidation products of aldehydes, are rarely detected in heteropteran secretions [5, 35], suggesting that oxidative reactions do not readily occur within the heteropteran secretory glands. Similarly, oxidation of unsaturated fatty acids in the glands, which generates aldehydes via cleavage of carbon–carbon bond, was not expected. The biosynthetic pathways of OHE and (*E*)‐2‐decenal in heteropteran species have not been fully identified; however, our findings suggest that OHE and (*E*)‐2‐decenal are synthesised by acetyl‐CoA‐dependent chain elongation or chain‐shortening pathway from saturated fatty acids, like palmitic acid, via β‐oxidation.

The observed incorporation of ^13^C‐labelled glucose into tridecane in *H*. *halys* is consistent with previous reports describing the *de novo* biosynthesis of odd‐chain hydrocarbons in insects and mites [29, 36]. This occurs via the reduction of fatty acyl‐CoA to its corresponding aldehydes, followed by decarboxylation (loss of CO_2_) [37, 38]. In some cases, myristyl aldehyde, a candidate precursor of tridecane biosynthesis, was detected in *H*. *halys* secretions. This study could not confirm whether ^13^C‐labelled glucose was incorporated into myristyl aldehyde due to its instability and scarcity. Similar enzymes may be responsible for the production of hydrocarbons by *H*. *halys*.

This study determined that dodecane in *H*. *halys* secretions is synthesised from glucose metabolites. If the hydrocarbons in *H*. *halys* are synthesised from fatty acids, the dodecane precursor may be an odd‐chain fatty acid. Odd‐chain fatty acids were not detected in *H*. *halys*. In the production of moth pheromone, C_19_ fatty acid is reported to be a precursor of even‐chain hydrocarbon [39]. The Baeyer–Villiger oxidation reaction converts C_18_ fatty acids in mites to C_17_ formate, which is one carbon shorter [40]. Accordingly, future research should explore the mechanisms underlying the production of the unique dodecane carbon skeleton in *H*. *halys*.

In this study, the fatty acid composition of the MTG complex of *H*. *halys* differed from that of the food sources (peanut seeds and carrots). This suggests that *H*. *halys* does not rely entirely on dietary fatty acids and can synthesise them as needed. This is supported by the ability of other heteropteran species to synthesise non‐essential fatty acids *de novo* [26]. Short‐chain aldehydes and hydrocarbons are *de novo* synthesised in *H. halys*. Given that these secretions play essential roles in chemical communication and defence in *H*. *halys*, it may be reasonable for this species to maintain the biosynthetic machinery of these secretions and allocate resources for *de novo* synthesis.

In conclusion, this study demonstrated that OHE, (*E*)‐2‐decenal, dodecane and tridecane, which are secretory components of *H*. *halys*, were synthesised *de novo* from dietary glucose. Myristic, stearic, palmitic, palmitoleic and oleic acids, but not linoleic acid, are synthesised *de novo* in *H*. *halys*. Furthermore, the composition of the fatty acid mixture isolated from the *H*. *halys* MTG complex differed from that isolated from food sources. This may be linked to the ability to synthesise or reallocate fatty acids and take them up from the diet.

## Conflict of interest

The authors declare no conflict of interest.

## Author contributions

HF and KN designed and performed the experiments, analysed the data and wrote the manuscript.

## Supporting information


**Fig. S1.** Morphology of *Halyomorpha halys* adult. (A) Ventral view of *H*. *halys* adult. Arrow shows the opening (orificium externum) from which the secretions are released. (B) Ventral view of the dissected *H*. halys adult. Arrow shows the metathoracic scent gland (MTG) complex.
**Fig. S2.** Gas chromatography/mass spectrometry analysis of methyl myristate in the metathoracic scent gland (MTG) complex of *Halyomorpha halys* reared with ^13^C‐labelled glucose. (A and B) Mass spectra of methyl myristate standard. (A′ and B′) Mass spectra of methyl myristate in the MTG complex of *H*. *halys* reared with ^13^C‐labelled glucose.

## Data Availability

The data supporting the findings of this study are available in the figures and Supporting Information. Raw data are available from the corresponding author (noge@akita-pu.ac.jp) upon request.
