# Supplementary material for: De novo synthesis of short‐chain aldehydes and hydrocarbons secreted by the brown marmorated stink bug Halyomorpha halys
Source: FEBS Open Bio. 2026 Jul 21:10.1002/2211-5463.70314. Online ahead of print. doi: 10.1002/2211-5463.70314 (PMC13398841; doi:10.1002/2211-5463.70314)
Supplement: Supplementary file 1 — Fig. S1. Morphology of Halyomorpha halys adult. (A) Ventral view of H. halys adult. Arrow shows the opening (orificium externum) from which the secretions are released. (B) Ventral view of the dissected H. halys adult. Arrow shows the metathoracic scent gland (MTG) complex. Fig. S2. Gas chromatography/mass spectrometry analysis of methyl myristate in the metathoracic scent gland (MTG) complex of Halyomorpha halys reared with 13C‐labelled glucose. (A and B) Mass spectra of methyl myristate standard. (A′ and B′) Mass spectra of methyl myristate in the MTG complex of H. halys reared with 13C‐labelled glucose. [file FEB4-9999-0-s001.pdf]

Supporting information for:

De novo synthesis of short-chain aldehydes and hydrocarbons secreted by the brown marmorated stink bug *Halyomorpha halys*

Haruna Fujimori and Koji Noge

Department of Biological Production, Akita Prefectural University, 241-438 Kaidobata-nishi, Shimoshinjyo-Nakano, Akita 010-0195, Japan

Correspondence: noge@akita-pu.ac.jp (K. N.)

Supplementary Figures

A B

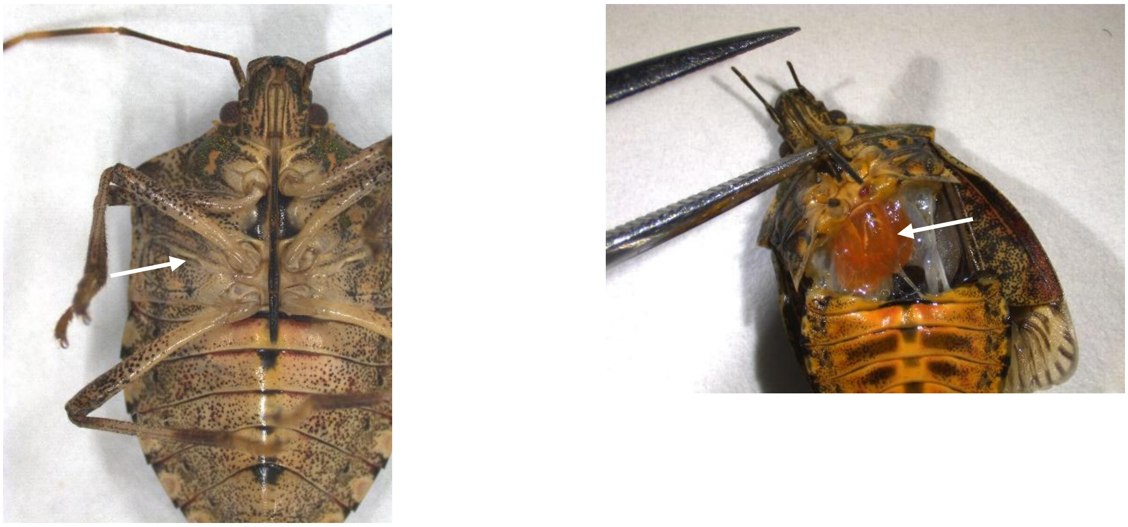

Fig. S1. Morphology of *Halyomorpha halys* adult. (A) Ventral view of *H*. *halys* adult. Arrow shows the opening (orificium externum) from which the secretions are released. (B) Ventral view of the dissected *H*. halys adult. Arrow shows the metathoracic scent gland (MTG) complex.

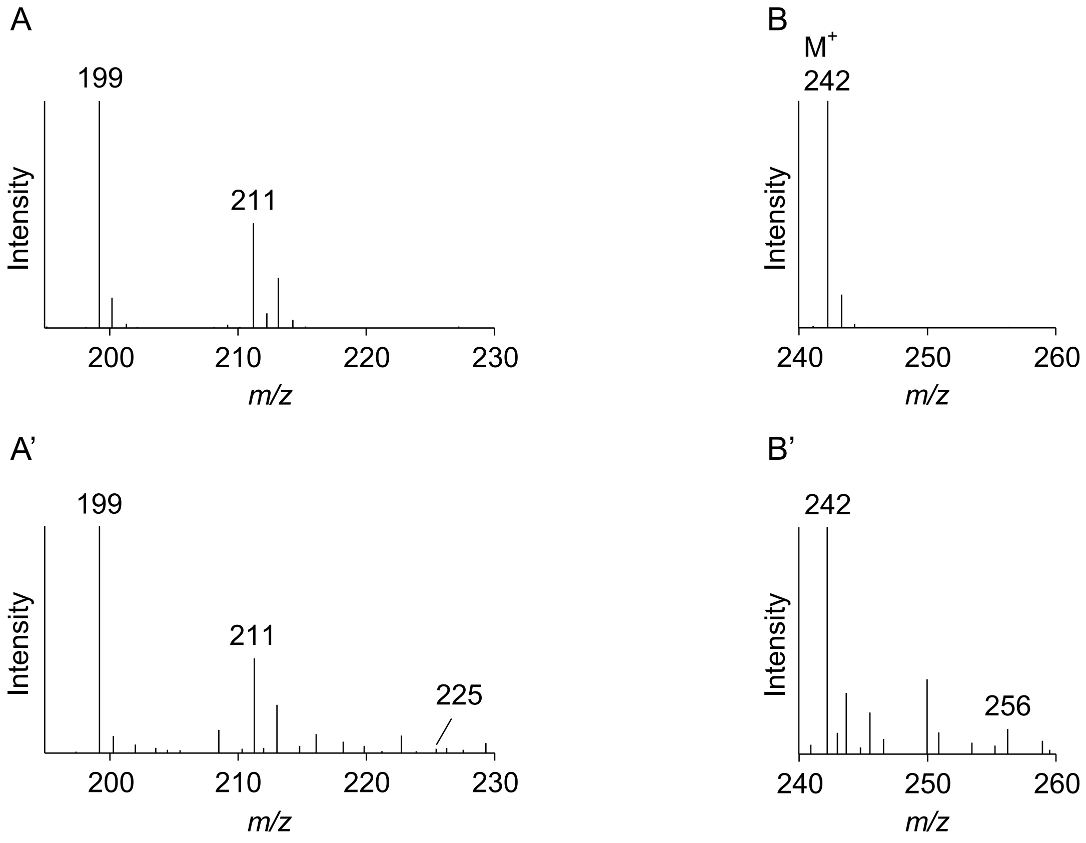

Fig. S2. Gas chromatography/mass spectrometry analysis of methyl myristate in the metathoracic scent gland (MTG) complex of *Halyomorpha halys* reared with ^{13}C-labelled glucose. (A and B) Mass spectra of methyl myristate standard. (A' and B') Mass spectra of methyl myristate in the MTG complex of *H. halys* reared with ^{13}C-labelled glucose.
